# Asynchronous Chirp Slope Keying for Underwater Acoustic Communication

**DOI:** 10.3390/s21093282

**Published:** 2021-05-10

**Authors:** Dominik Jan Schott, Andrea Gabbrielli, Wenxin Xiong, Georg Fischer, Fabian Höflinger, Johannes Wendeberg, Christian Schindelhauer, Stefan Johann Rupitsch

**Affiliations:** 1 Department of Microsystems Engineering (IMTEK), University of Freiburg, 79110 Freiburg, Germany; and.gabbrielli@gmail.com (A.G.); fabian.hoeflinger@imtek.uni-freiburg.de (F.H.); stefan.rupitsch@imtek.uni-freiburg.de (S.J.R.); 2 Department of Computer Science (IIF), University of Freiburg, 79110 Freiburg, Germany; xiongw@informatik.uni-freiburg.de (W.X.); wendeber@informatik.uni-freiburg.de (J.W.); schindel@informatik.uni-freiburg.de (C.S.); 3 Fraunhofer EMI, 79588 Efringen-Kirchen, Germany; Georg.Fischer@emi.fraunhofer.de

**Keywords:** underwater communication, wireless communication, acoustic communication, ultrasound acoustics, digital signal processing, chirp modulation, chirp slope keying, chirp spread spectrum

## Abstract

We propose an asynchronous acoustic chirp slope keying to map short bit sequences on single or multiple bands without preamble or error correction coding on the physical layer. We introduce a symbol detection scheme in the demodulator that uses the superposed matched filter results of up and down chirp references to estimate the symbol timing, which removes the requirement of a preamble for symbol synchronization. Details of the implementation are disclosed and discussed, and the performance is verified in a pool measurement on laboratory scale, as well as the simulation for a channel containing Rayleigh fading and Additive White Gaussian Noise. For time-bandwidth products (TB) of 50 in single band mode, a raw data rate of 100 bit/s is simulated to achieve bit error rates (BER) below 0.001 for signal-to-noise ratios above −6 dB. In dual-band mode, for TB of 25 and a data rate of 200 bit/s, the same bit error level was achieved for signal-to-noise ratios above 0 dB. The simulated packet error rates (PER) follow the general behavior of the BER, but with a higher error probability, which increases with the length of bits in each packet.

## 1. Introduction

In air, there is a plenitude of electromagnetic wave communications available to connect devices, but due to their strong fading in the underwater channel, acoustic communication has shown beneficial results. While there are several communications systems for deep open water communications available, where offshore industries and naval warfare have accelerated technological advancement, shallow water still challenges communication attempts after over a hundred years of research [1,2,3]. This may stem from the strongly selective frequency fading, high phase noise and fast echoes and for moving nodes, due to a strong Doppler effect which characterizes the instability of the acoustic underwater channel [4,5,6,7,8,9]. The shallower the channel is, the more pronounced this inhibitions become. Previous investigations into the field of acoustic underwater communications have shown promising results [10,11,12], but concentrated on deeper bodies of water over longer distances of several kilometer in audible or sub-audible frequencies [13,14]. While high-bandwidth communication with large spectral efficiencies will be prone to upset the natural habitat if performed in the audible range of the maritime fauna [15,16], narrow-band methods as commonly found in frequency-shift keying (FSK) modems [17,18] are vulnerable to the fading effects discussed before.

The ongoing interest is a result of the strong attenuation of radio signals underwater and the long distances that require to be covered. Application examples, where underwater acoustic communication is crucial are diver tracking [19,20], robot/autonomous underwater vehicle (AUV) telemetry [21,22,23,24], and underwater sensor networks [25,26].

### 1.1. Historical Overview

Evidence suggests that the general idea to sweep the frequency of a carrier to transmit information may be as old as (breathing) life on earth [27], but the first modern record we found is Hüttmann et al.’s patent for a distance measurement method from 1940 [28]. The idea to use chirps for modulating a signal, e.g., as Chirp Slope Keying (CSK) is often attributed to Winkler et al.’s work in the early 1960s [29], and also sometimes referred to as linear frequency modulation (LFM) [30]. The concept although was patented as early as 1949 by Darlington et al. [31]. After the application in satellite radio transmission during the Cold War era [32,33], the interest in chirp modulations mostly vanished due to the low achievable data rates. For radio communications, this changed in 2013 with the establishment of the LoRa protocol as a low-power long range radio modulation for small consumer applications [34,35].

### 1.2. Research Problem

We investigate a modulation scheme that is intended for short messages, which in our application of diver status and localization packages are intended to carry an unique ID and a short amount of status information, as well as limited telemetric data, similar to the protocol of the AHOI modem [18]. We found in our previous investigations [36] that conventional chirp slope keying demodulation approaches, e.g., as proposed in the works of [8,23,37,38], show a critical flaw for the application in the transmission of very short multi-band packets, where the probability that a single channel is comprised entirely of one symbol state is high. This leads to one of the matched filter outputs having only the low-valued crosscorrelation of up vs. down chirp to estimate the symbol timing. One common solution is in the use of well known preambles, that feature an adequate amount of all states in all bands, to ensure symbol synchronization, with the draw-back that precious time slots in the channel are occupied by the overheads. Another solution is to implement a coding scheme, which avoids patterns that lead to single channel featuring only a single symbol state. Furthermore, the target application of our communication scheme in diver or UAV communication implicates an underwater environment, where reverberating surfaces are close-by, e.g., the water surface as well as a ship’s hull in case of maintenance divers or the ground for scientific UAVs. Therefore, the assumption of semi-infinite bodies of water does not hold. We emulate this environment by using a small pool for our measurements, as shown in Section 2.5, which introduces strong reverberations from multi-path propagation. Finally, we simulate the overall BER for an idealized channel in Section 4 to provide performance estimators, which can be compared to other systems, along with the PER that is often neglected in other works.

### 1.3. Related Work

Chirp modulation of acoustic signals under water have gathered attention since the early 2000s [39], and is researched continuously by groups around the world ever since. While the achievable data rate is severely limited compared to narrow-band schemes [5], CSK will be especially of interest, when the data amount is low and the channel inhibitions are strong. Kaminsky and Simanjuntak [37] present a performance evaluation of CSK in additive white Gaussian noise (AWGN) environments and for more realistic underwater models. Lee et al. propose similar methods for acoustic aerial communications (AAC). A low-cost underwater acoustic modem is proposed by Benson et al. [17], which uses FSK instead of CSK. Symbol synchronization is a large issue underwater due to Multi-path and Doppler effects, He et al. [40] therefore propose a self-synchronization method for CSK-type communication. Demirors and Melodia [41] subjoin methods of code division multiplexing to chirp communication to enhance stealthiness. The Fractional Fourier Transform (FrFT) is employed by Yuan et al. [42] to enable a multiuser communication system. Khyam et al. [20] iterate on the multiuser possibility by proposing several sets of orthogonal chirp waveforms. Interference cancellation is crucial for underwater communication, this topic has been addressed by Diamant [43]. In the more recent work of Lee et al. [44], the authors explore the parameter space of chirp spread spectrum (CSS) methods in context of long range communication.

The performance of our approach in this work has partially been reported in [36]. The scope of this contribution is to report on the details of our system’s inner structure and underlying algorithms. This investigation zooms in on the modulation and demodulation part of typical basic elements of digital communication systems [45], hence, expects coded input and will return still coded output. Consequently, additional forward error correction coding is likely to improve the estimation of the overall system [46], but is not part of this investigation.

## 2. Materials and Methods

We propose a different approach and overcome the demodulators synchronization vulnerability described in Section 1.2 by analyzing the sum of both matched filter outputs for up and down chirps. The resulting superposed signal always features an autocorrelation peak for each symbol, which is then used to retrieve the signal differences between the matched filter outputs to determine the symbol state. We discuss this aspect in detail in Section 2.4. In the following we describe the structure of our communication signal chain in detail, to embed our contribution to the demodulation process properly and fully disclose our method for ease of comparison.

### 2.1. Basic System Structure

**Figure 1:**
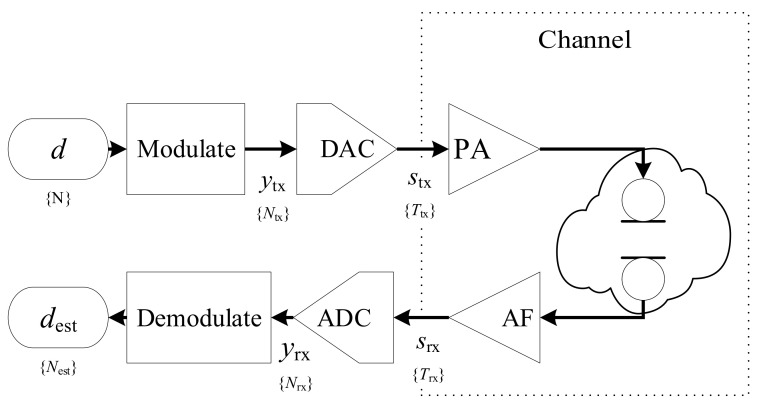
Flow diagram of the basic communication chain: The data *d* is modulated, amplified before transmission, filtered and amplified at reception, and demodulated as $d_{\mathrm{est}}$. The entire analog domain is regarded as part of the communication channel.

Our system is divided into functional blocks with the modulation and demodulation in focus, as shown in Figure 1; more details about the structure inside those two blocks is discussed in Section 2.2 and Section 2.4.

The data *d* of length *N* is modulated into the digital sequence of linear up and down chirps $y_{\mathrm{tx}}$, now of length $N_{\mathrm{tx}}$, as illustrated in Figure 1. The DAC then converts this into the the output $s_{\mathrm{tx}}$, which is an analog continuous real-valued signal of length $T_{\mathrm{tx}}$ that is boosted by a power amplifier (PA) before it is turned into an acoustic wave by a piezoelectric transducer.

The received signal is bandpass-filtered and amplified by an analog active filter (AF) into the continuous real-valued and band-limited received signal $s_{\mathrm{rx}}$ of length $T_{\mathrm{rx}}$. The ADC samples the received signal into the sequence $y_{\mathrm{rx}}$ of length $N_{\mathrm{rx}}$. The demodulation step estimates the originally sent sequence as $d_{\mathrm{est}}$ given a small set of prior information about the original signal, e.g., the ideal chirp parameters.

We consider exclusively time discrete signals throughout this work, sampled at points

(1) $$n=\left\lfloor t\;f_{s}\right\rfloor \in Z,$$

where *t* is the time of observation and $f_{s}$ the sampling frequency. For simplicity we assume the sampling frequency of transmitter and receiver to be equal, save an oscillator frequency mismatch of $\Delta f_{s}$ and phase offset of $\Delta \phi_{s}$. In practical application, this is not required, but only the parameters that describe the used waveform sufficiently.

### 2.2. Modulation

**Figure 2:**
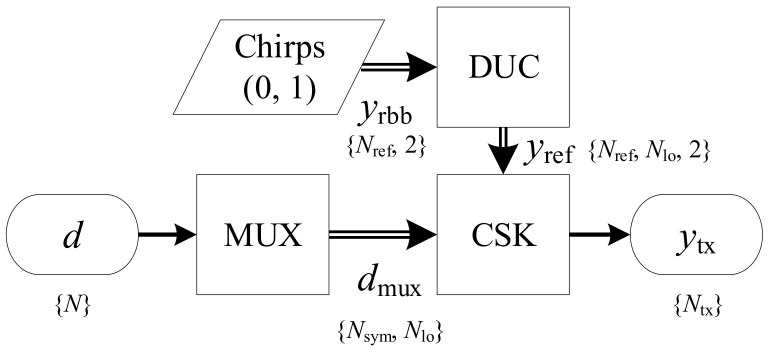
Modulator block in detail: The data input *d* is mapped onto $N_{\mathrm{lo}}$ sub-bands through a multiplexer (MUX) and modulated by the up-converted chirped symbols from the DUC. The transmission sequence $y_{\mathrm{tx}}$ is assembled by the CSK block, already in the transmission band. Simple arrow lines indicate vectors, double lines arrays.

Before transmission, data *d* is multiplexed onto $N_{\mathrm{lo}}$ sub-bands of information. The $N_{\mathrm{lo}}$ sub-bands are then modulated through the Chirp Slope Keying (CSK) block. The slope sign of the reference up and down chirps are up-converted by the Digital Up-Converter (DUC) into the transmission bands, see Figure 2. The DUC is often omitted in acoustic communication due to the relatively low frequency of the transmission bands compared to radio communication, but allows for a more efficient use of storage and reduces the computational effort, which is why we include it.

#### 2.2.1. Linear Chirp Creation

Initially, a reference chirp $y_{\mathrm{rbb}}$ is generated through

(2) $$\begin{array}{cc} y_{\mathrm{rbb}}\left[n\right] & =\left\{\begin{array}{cc} w\left[n\right]\;\sin\left(\varphi \left[n\right]\right), & \mathrm{for}\;0<n\leq N_{\mathrm{ref}}\\ 0, & \mathrm{else}. \end{array}\right. \end{array}$$

For simplicity of calculation we normalize (note that implementations in Matlab often use the Nyquist frequency $f_{\mathrm{nyq}}=f_{s}/2$ for normalization instead) the angular frequencies

(3) $$\begin{array}{cc} \omega_{0} & =2\pi f_{0}/f_{s},\;\mathrm{and}\\ \omega_{1} & =2\pi f_{1}/f_{s} \end{array}$$

with the start frequency $f_{0}$ and the stop frequency $f_{1}$. This allows the definition of the argument $\varphi \left[n\right]$ for a linear chirp as

(4) $$\omega \left[n\right]=\omega_{0}+n\;\frac{\omega_{1}-\omega_{0}}{N_{\mathrm{ref}}},$$

and therefore

(5) $$\varphi \left[n\right]=\varphi_{0}+\int_{0}^{n}\omega \left[\nu \right]d\nu .$$

With (4) and (5), we can calculate the instantaneous phase for a linear sinusoidal chirp according to

(6) $$\varphi \left[n\right]=\varphi_{0}+n\;\omega_{0}+\frac{1}{2}n^{2}\;\frac{\omega_{1}-\omega_{0}}{N_{\mathrm{ref}}}.$$

The modulation will become clearer if we substitute

(7) $$\omega_{c}=\frac{\omega_{1}+\omega_{0}}{2},\;$$

(8) $$B_{\omega }=\left|\omega_{1}-\omega_{0}\right|,$$

and introduce

(9) $$\zeta =\mathrm{sign}\left(\omega_{1}-\omega_{0}\right).$$

**Figure 3:**
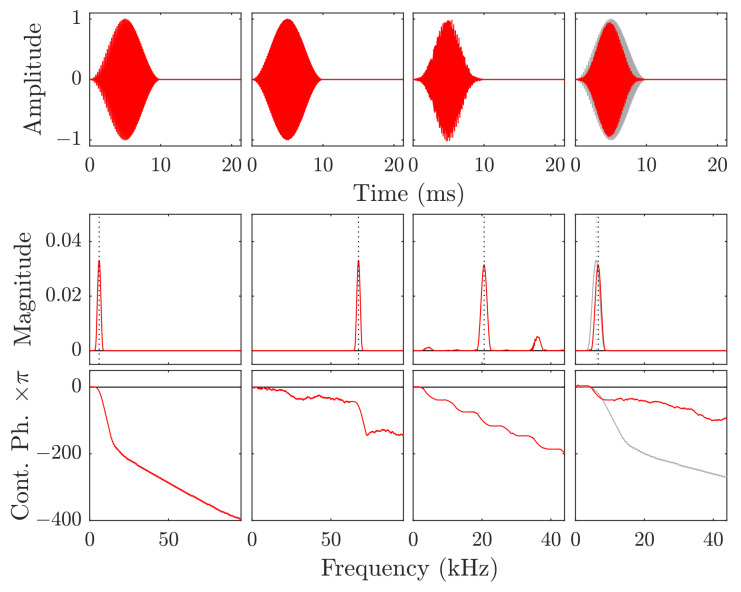
Resampling example for a linear chirp with $f_{c}=3\;\mathrm{kHz}$, $B_{f}=5\;\mathrm{kHz}$, and T=10 ms. **leftmost:** Base band signal $y_{\mathrm{tb}}$ at the transmitter, **center left:** Transmission band $y_{\mathrm{ib}}$, **center right:** Undersampled signal on reception, **rightmost:** Down-converted base band signal $y_{\mathrm{bb}}$ at the receiver where the originally transmitted signal is overlayed in gray. The transmission band’s center frequency is at 67.5 kHz. In the experiments, the intermediate band on reception is around 20.5 kHz due to undersampling with only $f_{s}=88\;\mathrm{kHz}$. Note the changed frequency scale for the Bode plots in the two columns on the right.

The instantaneous phase of the chirp from (6) then takes the form of

(10) $$\begin{array}{cc} \varphi \left[n\right]= & \varphi_{0}+n\;\omega_{c}+\zeta \frac{B_{\omega }}{2}n\left(1+\frac{n}{N_{\mathrm{ref}}}\right), \end{array}$$

where each bit of the data is mapped onto the sign ζ. The resulting chirp sequence is generated in the base-band frequencies according to (2) through Algorithm A1 and up-converted to each channel through Algorithm A3. In Figure 3 (leftmost), an example for such a base-band chirp is shown and the result of the up-conversion in Figure 3 (center left).

For a fixed transmission channel communication, the up-conversion through a DUC can be omitted and the reference chirps directly be calculated in the transmission band, but for the sake of flexibility, we added the up-conversion as a separate block. The resulting single chirp sequences $y_{\mathrm{ref}}$ for all $N_{\mathrm{lo}}$ transmission channels can be stored permanently and only requires to be recalculated, if the parameters, e.g., sampling frequency $f_{\text{s}}$, chirp length $N_{\mathrm{ref}}$, side-band center frequency $f_{\mathrm{ch}}$ or bandwidth *B* change. While intuitively both chirp slope sequences may be pre-generated, we omit this redundancy on implementation as the inverse slope sign is equivalent to a time reversal of the entire chirp sequence.

#### 2.2.2. Shaping

The amplitude shaping window $w\left[n\right]$ restricts the sequence to be non-zero in the interval between 0 and $N_{\mathrm{ref}}$ only. While this can be achieved through different window functions, the tapered cosine, i.e., Tukey window is used in this work, because it can be varied easily between the rectangular, i.e., Dirichlet window and a sine, i.e., Hann window, by changing the single tuning factor $a_{t}$ to 0 or 1, respectively [47]. This sets the main lobe width between $4\pi /N_{\mathrm{ref}}$ and $8\pi /N_{\mathrm{ref}}$, as well as the peak sidelobe between −13 dB and −31 dB [48]. The Tukey window is mathematically defined as [48]

(11) $$w\left[n\right]=\left\{\begin{array}{cc} \frac{1}{2}\left[1-\cos\left(\frac{\pi }{N_{\mathrm{tk}}}n\right)\right], & \mathrm{for}\;0<n\leq N_{\mathrm{tk}}\\ 1, & \mathrm{for}\;N_{\mathrm{tk}}<n\leq \left(N_{\mathrm{ref}}-N_{\mathrm{tk}}\right)\\ \frac{1}{2}\left[1-\cos\left(\frac{\pi }{N_{\mathrm{tk}}}\left(n-\left(N_{\mathrm{ref}}-N_{\mathrm{tk}}\right)\right)\right)\right], & \mathrm{for}\;\left(N_{\mathrm{ref}}-N_{\mathrm{tk}}\right)<n\leq N_{\mathrm{ref}}\\ 0, & \mathrm{otherwise}, \end{array}\right.,$$

where the threshold of the taper is set by

$$N_{\mathrm{tk}}=\frac{a_{t}N_{\mathrm{ref}}}{2}.$$

**Figure 4:**
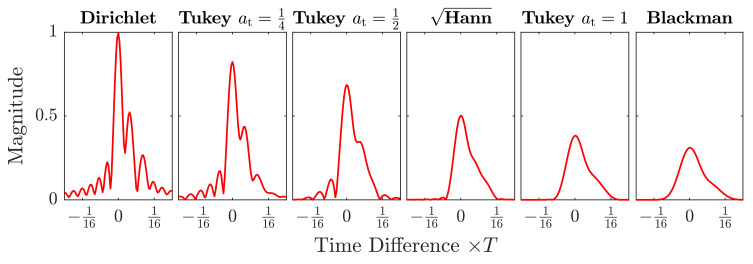
Autocorrelation magnitude comparison of a selection of shaping window functions. All magnitudes are normalized by the Dirichlet shaped chirp power for comparison. Gaussian noise was added to a signal-to-noise ratio SNR=0 dB, as well as two echoes at n=9 and n=19. To the left the spatial resolution and peak power is higher, to the right the inter-signal interference and spectral leakage are reduced.

**Figure 5:**
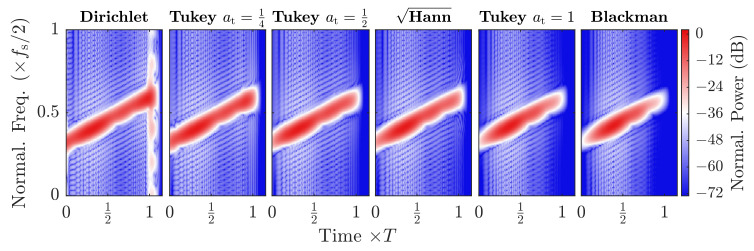
Simulated spectrograms of the autocorrelations of a selection of shaping window functions. All magnitudes are normalized by the Dirichlet shaped chirp power for comparison. Gaussian noise was added to a signal-to-noise ratio of 0 dB, as well as two echoes at n=9 and n=19.

The amplitude shape of the chirp can thus be adapted to the channel, depending on the application, i.e., if a narrow autocorrelation peak width is required for spatial distinction of two close reverberations or wide smooth peaks are desired for more robust communication. The implementation we used in this work is described in Algorithm A2. A simplified comparison of a selection of window functions for a close echo is shown in Figure 4 and Figure 5.

#### 2.2.3. Input Multiplexing

When data *d* of length *N* is submitted for transmission, the 1-D binary sequence is first multiplexed onto $N_{\mathrm{lo}}$ channels through

(12) $$\begin{array}{cc} d_{\mathrm{mux}}\left[k,\;l\right] & =d\left[n\right],\;\mathrm{where}\\ n & =\;\left(k-1\right)\;N_{\mathrm{lo}}+l,\\ k & \in \;\left[1,\;N_{\mathrm{sym}}\right],\\ l & \in \;\left[1,\;N_{\mathrm{lo}}\right]. \end{array}$$

The multiplexed sequence length $N_{\mathrm{sym}}$ is

(13) $$N_{\mathrm{sym}}=\left\lfloor \frac{N}{N_{\mathrm{lo}}}\right\rfloor ,$$

therefore *N* needs to be an integer multiple of $N_{\mathrm{lo}}$. To assert this, we use a simple zero-padding algorithm. If bits are added, they will remain in the data on reception and need to be removed on a higher level later on. This can be avoided, by matching the data length to the desired number of channels in advance, but for a more flexible and general approach, we implement the zero-padding approach and truncate to byte-sizes of 8.

#### 2.2.4. Chirp Slope Keying

A fast way to modulate the binary sequence is to have a simple decision block, that will output a chirp sequence of either upward or downward slope according to the binary value at the input (see Algorithm A5). The segments of the ouput sequence $y_{\mathrm{tx}}$ are assembled by filling each interval of length $N_{\mathrm{ref}}$ with the normalized sum of the superposed channels’ sequences to

(14) $$y_{\mathrm{tx}}\left[n\right]=\frac{1}{N_{\mathrm{lo}}}\sum_{l}^{N_{\mathrm{lo}}}y_{\mathrm{ref}}\left[n,\;l,\;d_{\mathrm{mux}}\left[k,\;l\right]\right].$$

The output sequence is transferred into an analog signal $s_{\mathrm{tx}}$, e.g., by a DAC, amplified by the PA and transmitted. Alternatively, this modulation can be calculated by zero-padding the multiplexed bit sequences $d_{\mathrm{mux}}$ by length $N_{\mathrm{ref}}$ and convolving the resulting sequence with the reference signal $y_{\mathrm{ref}}$, but this approach is neither efficient in memory usage, nor the calculation steps required [49].

### 2.3. Channel Model

We adapted the simulation approach from [37] to estimate the performance of the modulation and demodulation modules in a controlled fashion. The channel model includes simplified Rayleigh fading that multiplies the signal amplitude by the magnitude of two independent, but identically distributed random processes

(15) $$\begin{array}{cc} A_{r} & =\;|\left(\mathrm{randn}\left\{N_{\mathrm{rx}}\right\}+i\;\cdot \;\mathrm{randn}\left\{N_{\mathrm{rx}}\right\}\right)\;\sigma_{r}|,\;\mathrm{where}\\ i & =\sqrt{-1}, \end{array}$$

with a distribution parameter $\sigma_{r}=1$. The random sequences are generated through the randn function of Matlab that generates a normally distributed random value. An AWGN in the form of

(16) $$\epsilon_{n}=\sigma_{g}\;\cdot \;\mathrm{randn}\left\{N_{\mathrm{rx}}\right\}$$

is used to model the thermal noise of the receiver.

For simulations, we assume the receiver samples a combination (15) and (16) with the transmitted signal, at a random packet reception time offset $n_{\tau }$, without additional reverberations from multiple paths

(17) $$y_{\mathrm{rx}}\left[n\right]=A_{r}\;y_{\mathrm{tx}}\left[n+n_{\tau }\right]+\epsilon_{n}.$$

### 2.4. Demodulation

**Figure 6:**
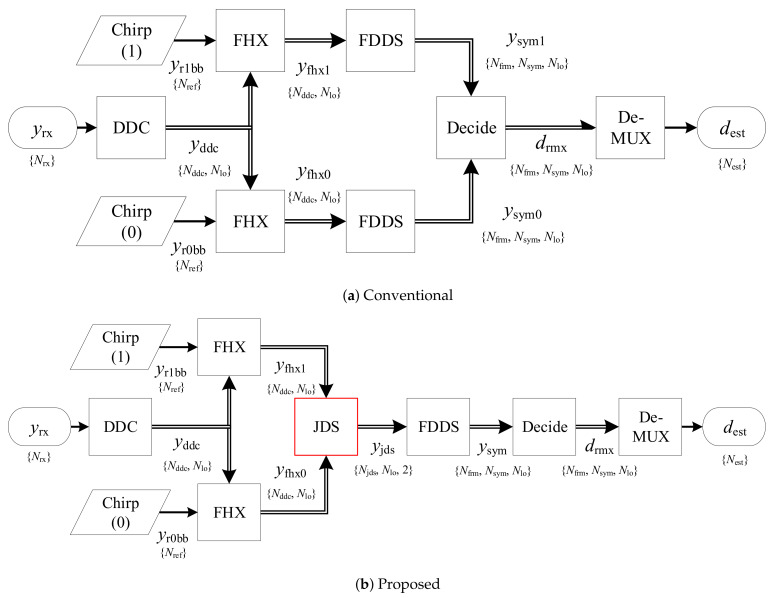
Comparison of the conventional and proposed demodulation as a block diagram in detail. (**a**) In the former case, the received sequence *y*_rx_ is processed by Digital Down-Coverter (DDC), compressed through a Fast Hilbert Cross-Correlator (FHX), converted into symbol space through Frame Detect & Downsample (FDDS), which is interpreted by a binary decision (Decide) block, and finally assembled into the estimated data output *d*_est_ through a reverting multiplexer (De-MUX). (**b**) We propose the insertion of a superposition in the compressed sample space through the Join & Downsample (JDS) block that creates a sum signal for symbol timing and a difference signal for symbol extraction.

The digitized signal $y_{\mathrm{rx}}$ is translated into the baseband for each of the $N_{\mathrm{lo}}$ channels in the Digital Down-Converter (DDC) block, as shown in Figure 6. The Fast Hilbert Cross-Correlator (FHX) block compresses the signal further into arrays $y_{\mathrm{fhx}}$ for additional dimensions for each of the reference chirps of both slope signs. The block Join & Downsample (JDS) attempts coherent addition and subtraction of the 2 signal arrays for each channel. The resulting sum and difference signals in in $y_{\mathrm{jds}}$ are analyzed by the Frame Detect & Downsample (FDDS) block and the input signal divided into separate frames $y_{\mathrm{sym}}$, now at symbol rate. The final decision block translates the symbols into binary values *d* and estimates the demodulation performance. Each block is described below in detail.

#### 2.4.1. Digital Down-Converter

Before the signal is fed into the resource intensive compression algorithm, we exploit the bandlimited nature of the signal and bring it down into the baseband, by calculating

(18) $$\begin{array}{cc} y_{\mathrm{tb}}\left[n,\;c\right]\;= & \;\mathrm{BPF}\left\{y_{\mathrm{rx}}\left[n\right]\right\},\\ y_{\mathrm{ib}}\left[n,\;c\right]\;= & \;y_{\mathrm{tb}}\left[n\right]\;y_{\mathrm{lo}},\\ y_{\mathrm{bb}}\left[n,\;c\right]\;= & \;\mathrm{LPF}\left\{y_{\mathrm{ib}}\left[n,\;c\right]\right\}, \end{array}$$

where the functions LPF denotes an arbitrary lowpass filter, and BPF any suitable bandpass filter. The implementation is attached in Algorithm A4. The signal content outside of the band is suppressed by the analog bandpass filter of the receivers signal conditioning before the sampling. This is especially important for undersampling a signal to limit the aliasing effect of noise. To achieve the downconversion we first multiply the bandpass filtered raw signal $y_{\mathrm{tb}}$ of each transmission band with sine waves $y_{\mathrm{lo}}$ of frequency $f_{\mathrm{lo}}$ to create the intermediate signal $y_{\mathrm{ib}}$. This operation shifts the content of each of the $N_{\mathrm{lo}}$ channels into the baseband, where a lowpass filter removes the higher harmonics and produces the baseband sequence $y_{\mathrm{bb}}$. In doing so, the memory consumption increases by the number of channels $N_{\mathrm{lo}}$, a one-dimensional real-valued input sequence of $\left[N_{\mathrm{rx}},1\right]$ gets mapped onto an $\left[N_{\mathrm{rx}},N_{\mathrm{lo}}\right]$ output array. The sequence can be truncated in frequency domain to an interval around the center, since most of the frequency bands ideally contain no information about the signal and one loses only information about noise and interference. We effectively resample the sequence to

(19) $$\begin{array}{cc} y_{\mathrm{ddc}}= & \;\mathrm{resample}\left\{y_{\mathrm{bb}},\;f_{s},\;f_{s1}\right\},\;\mathrm{where}\\ f_{s1}= & \;\frac{f_{s}}{N_{\mathrm{res}1}}. \end{array}$$

As we use the single sideband approach, a minimal interval is limited by the center frequency

(20) $$f_{c}=\frac{1}{2}\left(f_{1}+f_{0}\right)$$

and half the bandwidth

(21) $$B_{f}=\left(f_{1}-f_{0}\right).$$

Assuming a sampling rate of, e.g., $f_{s}=88\;\mathrm{kHz}$, a bandwidth of $B_{f}=2.5\;\mathrm{kHz}$ and a sub-band center frequency of $f_{c}=3.0\;\mathrm{kHz}$ as used in the dual-band case, the minimal one-sided base band is

(22) $$B_{\mathrm{fbbm}}=f_{c}+\frac{1}{2}B_{f},$$

which is for the given example $B_{\mathrm{fbbm}}=4.25\;\mathrm{kHz}$. Considering the original sample bandwidth and unchanged frequency bin width, the computation is reduced to $B_{\mathrm{fbbm}}/\frac{1}{2}f_{\text{s}}$, here by about 90% at most. The minimal interval truncation also removes information about the noise, so a trade-off is feasible that implements a larger interval of several bandwidths. Moreover, the whole band-shifting and resampling can effectively be done in the frequency domain with a shift and truncate operation, as described in detail in Algorithm A4. An example for a result of this operation is shown in Figure 3.

#### 2.4.2. Pulse Compression by Fast Hilbert Cross-Correlation

If time and magnitude of a received chirp are of interest, the calculation of the analytic signal after pulse compression through a matched filter is convenient. Hence, the next signal processing step is to convolve (operator ⊛) the received signal with the matched filter for both chirp slope signs

(23) $$\begin{array}{c} y_{\mathrm{mf}↑}=y_{\mathrm{ddc}}⊛y_{\mathrm{rbb}↑},\\ y_{\mathrm{mf}↓}=y_{\mathrm{ddc}}⊛y_{\mathrm{rbb}↓}, \end{array}$$

This increases the memory allocation to $\left[N_{\mathrm{rx}},N_{\mathrm{lo}},2\right]$ samples, as the downsampled sequences are compressed by both, up and down chirps. In case more different chirps are used, this increases the added dimension accordingly. The compressed pulse’s envelope is then calculated as the analytic signal through the norm of the signal and its Hilbert transform

(24) $$y_{\mathrm{fhx}}=\sqrt{y_{\mathrm{mf}}^{2}+H{\left\{y_{\mathrm{mf}}\right\}}^{2}}.$$

The calculations, both, matched filtering and envelope extraction, are performed in the frequency domain for convenience. After the Fourier transformation of the raw signal, we perform a bin-wise multiplication against the complex conjugated reference signals to obtain the compressed signals for both up and down chirps.

#### 2.4.3. Join & Downsample

Frame detection and symbol decision require information about the compressed pulse peak positions in time, which are difficult to establish in one matched filter branch, e.g., only the up chirp compression result, as there may be no peaks present, if the signal hypothetically only consists of down chirps. The JDS block first resamples the sequence to an integer fraction by $N_{\mathrm{res}2}$ to the sample rate

(25) $$\begin{array}{cc} y_{\mathrm{res}2}= & \;\mathrm{resample}\left\{y_{\mathrm{fhx}},\;f_{s1},\;f_{s2}\right\},\;\mathrm{where}\\ f_{s2}= & \;\frac{f_{s1}}{N_{\mathrm{res}2}},\\ N_{\mathrm{jds}}= & \;\left\lfloor \frac{N_{\mathrm{fhx}}}{N_{\mathrm{res}2}}\right\rfloor , \end{array}$$

then creates the sum and difference

(26) $$\begin{array}{cc} y_{\mathrm{sum}}\left[n,\;c\right] & =y_{\mathrm{fhx}↑}\left[n,\;c\right]+y_{\mathrm{fhx}↓}\left[n,\;c\right],\\ y_{\mathrm{dif}}\left[n,\;c\right] & =y_{\mathrm{fhx}↑}\left[n,\;c\right]-y_{\mathrm{fhx}↓}\left[n,\;c\right]. \end{array}$$

This operation requires coherence, since a phase difference between the up and down chirp compressed sequences leads to sub-optimal symbol detection. This condition will be fulfilled only if no Doppler shift is present, so sender and receiver do not move relative to each other [50]. For this work, we exclusively considered stationary conditions. The sum and difference sequences are stored in a joint array $y_{\mathrm{jds}}$ of size $\left\{N_{\mathrm{jds}},\;N_{\mathrm{lo}},\;2\right\}$.

#### 2.4.4. Frame Detect & Downsample

The FDDS block first estimates the frame positions in half symbol space, then uses this information to estimate the symbol phase of each frame and downsample it to full symbol space. First, we assume a known symbol length $N_{\mathrm{ch}2}$ from the reference chirp sequence and estimate it simply to

(27) $$N_{\mathrm{ch}2}=\left\lfloor T\;f_{s2}\right\rfloor =\left\lfloor N_{\mathrm{ref}}\;\frac{f_{s2}}{f_{s1}}\right\rfloor .$$

The mean magnitude of each of the $M_{H}$ half symbol frames of length $N_{H}$, where

(28) $$N_{H}=\left\lfloor \frac{N_{\mathrm{ch}2}}{2}\right\rfloor ,$$

is then calculated by only regarding the superposed pulses of both channels, which guarantees the presence of an autocorrelation peak in each symbol. Therefore, we calculate

(29) $$\begin{array}{cc} y_{\mathrm{ms}}\left[m\right]= & \;\sum_{n}^{N_{H}}y_{\mathrm{sum}}\left[n+\left(m-1\right)\right]\;N_{H},\;\mathrm{where}\\ m\in & \left[1,\;M_{H}\right],\\ M_{H}= & \;\left\lfloor \frac{N_{\mathrm{jds}}}{N_{H}}\right\rfloor , \end{array}$$

which reduces strong magnitude fluctuations before the data frame detection and resamples the sequence to half symbol space. As the envelope detection is very sensitive to non-steady slopes, we apply an additional 10th order lowpass filter

(30) $$y_{\mathrm{mLP}}=\mathrm{LPF}\left\{y_{\mathrm{ms}}\right\},$$

with an estimated cutoff frequency

(31) $$\begin{array}{cc} \omega_{\mathrm{MLP}}= & \;\frac{m_{\mathrm{MLP}}}{M_{H}},\;\mathrm{where}\\ m_{\mathrm{MLP}}= & \;\arg\max\left\{|\mathrm{FFT}\left\{y_{\mathrm{ms}}\right\}|\right\}. \end{array}$$

The frame detection algorithm has two parts. Initally, a threshold is calculated for the whole received sequence of each channel, then a state machine iterates through it and extracts frame start and end times. We estimate the threshold $y_{\mathrm{th}}$ by a simple clustering, that first calculates the mean amplitude of the lowpass filtered half symbol magnitude

(32) $${\bar{y}}_{\mathrm{mLP}}=\mathrm{mean}\left\{y_{\mathrm{mLP}}\right\},$$

then calculates the cluster means for both sides of the mean level,

(33) $$\begin{array}{c} {\bar{y}}_{\mathrm{mS}}=\mathrm{mean}\left\{y_{\mathrm{mLP}}\left[y_{\mathrm{mLP}}>{\bar{y}}_{\mathrm{mLP}}\right]\right\},\\ {\bar{y}}_{\mathrm{mN}}=\mathrm{mean}\left\{y_{\mathrm{mLP}}\left[y_{\mathrm{mLP}}<{\bar{y}}_{\mathrm{mLP}}\right]\right\}, \end{array}$$

where the lower mean value ${\bar{y}}_{\mathrm{mN}}$ is considered the noise level and the upper mean ${\bar{y}}_{\mathrm{mS}}$ the signal level. The threshold is then simply the arithmetic mean of those two levels

(34) $$y_{\mathrm{mTh}}=\frac{\left({\bar{y}}_{\mathrm{mS}}+{\bar{y}}_{\mathrm{mN}}\right)}{2}.$$

Subsequently, the state machine iterates through the sequence $y_{\mathrm{mLP}}$ and records an upwards slope if there are $M_{\mathrm{HL}}$ of samples below the threshold $y_{\mathrm{mTh}}$ followed by $M_{\mathrm{HH}}$ samples above it. We set both intervals as $M_{\mathrm{HL}}=M_{\mathrm{HH}}=2$, limiting the miminal frame size to $M_{\mathrm{HL}}+M_{\mathrm{HH}}-1=3\;\mathrm{samples}$. A state variable will keep track if the iteration is inside a frame and stores start index $m_{0}\left[p\right]$ and end index $m_{1}\left[p\right]$ of each *p*th frame. The frame limits are then reconstructed in sample space through scaling the indice by $M_{H}$,

(35) $$\begin{array}{cc} n_{0}\left[p\right] & =m_{0}\left[p\right]\;N_{H},\;\mathrm{and}\\ n_{1}\left[p\right] & =m_{1}\left[p\right]\;N_{H}. \end{array}$$

The single frames in sample space are then defined as

(36) $$\begin{array}{cc} y_{\mathrm{fSum}}\left[p,\;n\right]= & \;y_{\mathrm{sum}}\left[n_{0}\left[p\right]+n\right],\;\mathrm{where}\\ y_{\mathrm{fDif}}\left[p,\;n\right]= & \;y_{\mathrm{dif}}\left[n_{0}\left[p\right]+n\right],\;\mathrm{where}\\ n\in & \;\left[1,\;N_{\mathrm{frm}}\left[p\right]\right], \end{array}$$

where $y_{\mathrm{sum}}$ and $y_{\mathrm{div}}$ are the two sub-arrays of $y_{\mathrm{jds}}$ and include all $N_{\mathrm{lo}}$ channels as an additional dimension, respectively. The indexing of the channel dimensions has been omitted for ease of reading. The number of samples in each frame is

(37) $$N_{\mathrm{frm}}\left[p\right]=\;n_{1}\left[p\right]-n_{0}\left[p\right].$$

The last part of the block selects each data frame in the sample space, searches for the optimal sample offset $n_{\mathrm{off}}$ to maximize the symbol power and assembles a frame in symbol space accordingly. We assemble the power matrix for each frame *p* and each channel by iterating through the phase sample by sample

(38) $$\begin{array}{cc} A_{y}\left[p,\;n\right]= & \;\sum_{k}^{K\left[n\right]}{\left(y_{\mathrm{fSum}}\left[p,\;n+\left(k-1\right)\;N_{\mathrm{ch}2}\right]\right)}^{2},\;\mathrm{where}\\ K\left[n\right]= & \;\left\lfloor \frac{N_{\mathrm{jds}}-n}{N_{\mathrm{ch}2}}\right\rfloor . \end{array}$$

The optimal sample offset $n_{\mathrm{off}}$ is then estimated to

(39) $$n_{\mathrm{off}}\left[p\right]=\arg\max_{n}\left\{A_{y}\left[p,\;n\right]\right\}.$$

We use this to assemble the block’s final three-dimensional output sequences $y_{\mathrm{sym}}$, that span the number of detected frames, in each of which the number of symbols, and a constant number of channels. Hence, occupy memory is of size $\left[N_{\mathrm{frm}},\;N_{\mathrm{sym}},\;N_{\mathrm{lo}}\right]$ as we decimate

(40) $$y_{\mathrm{sym}}\left[p,\;k\right]=y_{\mathrm{fDif}}\left[p,\;n_{\mathrm{off}}\left[p\right]+\left(k-1\right)\;N_{\mathrm{ch}2}\right],$$

again the indexing for all channels is omitted.

#### 2.4.5. Symbol Decision

The symbol decision iterates through each frame’s symbol space difference sequence $y_{\mathrm{sym}}$ similarly to (32) to (34) of Section 2.4.4, by separating each frame in two clusters split by the mean symbol amplitude, and estimates the half distance between both clusters’ means as a threshold $y_{\mathrm{fTh}}$ for symbol decision for each channel. The decision equation is, therefore, simply

(41) $$d_{\mathrm{rmx}}\left[k\right]=\left\{\begin{array}{cc} 1, & \mathrm{for}\;y_{\mathrm{sym}}\left[k\right]>y_{\mathrm{fTh}}\\ 0, & \mathrm{otherwise}, \end{array}\right.$$

for the kth symbol of each channel and frame.

#### 2.4.6. De-Multiplexing

The last block of the demodulation chain re-assembles the $N_{\mathrm{lo}}$-dimensional symbol sequences of each frame into a one-dimensional bit sequence. The length of the received bit sequence $N_{\mathrm{est}}$ is first truncated to multiples of 8, as the application is meant to send and receive data bytewise, hence

(42) $$N_{\mathrm{est}}=8\;\left\lfloor \frac{N_{\mathrm{lo}}\;N_{\mathrm{sym}}}{8}\right\rfloor .$$

The data is then de-multiplexed by reshaping the sequences $d_{\mathrm{rmx}}$ with *n* in the range $\left[1,\;N_{\mathrm{est}}\right]$ to

(43) $$\begin{array}{cc} d_{\mathrm{est}}\left[n\right] & =d_{\mathrm{rmx}}\left[k,l\right],\;\mathrm{where}\\ k & =\frac{n}{N_{\mathrm{lo}}},\\ l & =n\;\mathrm{mod}\;N_{\mathrm{lo}}. \end{array}$$

### 2.5. Experimental Set-Up

**Figure 7:**
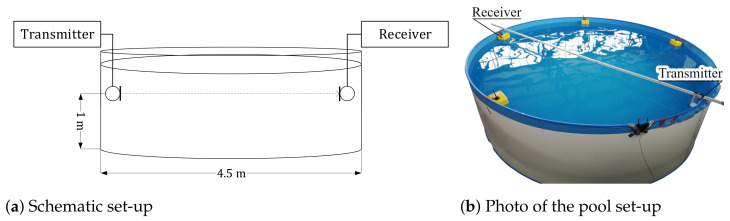
Schematic experimental set-up in for the acoustic transmission inside a water tank. The tank is filled with fresh water and located inside a closed building. A comparable scenario would be two divers working on a ship’s hull or an UAV inspecting a lake harbor’s foundations.

We conducted two experimental runs to verify our approach. One of a single band transmission, the other of a dual-band transmission. The experiments were performed in a steel-walled pool as shown in Figure 7, which was assembled temporarily inside a building. The transmitter and receiver hardware is a modified version of the indoor localization system [51], as we published before [19,36].

#### 2.5.1. Frequency Band Considerations

Acoustic underwater communication influences the maritime habitat, hence system and acoustic experiment designs have to minimize the interruption of natural communication [52] and ideally avoid mimicking animal calls (compare [53]). Fish and sharks have no hearing sensitivity for frequencies far above 10 kHz [15,52]. Sea mammals, e.g., dolphins, seals, and whales on the other hand are highly sensitive to frequencies of up to 150 kHz [54,55,56,57,58]. The general structure of sea mammal’s sound creation and hearing is known from anatomic considerations and follows similar mechanisms, with some whales having acoustic matching melons in their frontal part of the head that also serve as an acoustic bandpass and lens [57,59]. From a behavioral point of view, seals and dolphins are highly relevant, as they are well researched and commonly found near harbors and shores. As a simplified design rule, we regard that seals’ hearing is sensitive to sound frequencies below 80 kHz, with already increased sound pressure level thresholds, i.e., decreased hearing sensitivity above 60 kHz [60]. For dolphins, this hearing threshold is approximately 200 kHz, with decreased sensitivities above 150 kHz [54,55,56,57]. For all practical purposes, it has to be assumed that every transmission will be audible to sea mammals in the vicinity and can cause potential harm or changes in behavior. Additionally, the attenuation of acoustic underwater waves exceeds 20 dB km^−1^ for those frequencies, limiting the spatial sphere of influence. A limiting factor for coastal applications is natural and artificial noise, e.g., from the surf and ship traffic, which we regard as Brownian noise decaying at about 18 dB per decade [16,61]. For our system, we limit the communication band therefore as in Table 1.

#### 2.5.2. Experiment Parameters

The sampling rate of our acquisition unit is limited to $f_{s}=88\;\mathrm{kHz}$. As a result, the received signal is undersampled, i.e., the Nyquist frequeny is below the transmission band. While this mixing operation generally results in a leakage of signal power, the band-limited nature of the chirp sequences and the low noise environment limit this aliasing effect. This band-limitation is ensured by an additional analog bandpass-filter. The chirp parameters are listed in Table 2 for the single band and dual-band transmission.

The symbol rate and occupied bandwidth of both transmissions is kept constant. Therefore, the single band signal has twice the TB compared to the dual-band one. This implicates the ratio of symbol energy to noise energy to double as well [62],

(44) $$\frac{E_{\mathrm{sym}}}{E_{n}}=T\;B\;\gamma ,$$

where γ is the signal-to-noise ratio of the received signal. The expected data rate on the other hand is halved, as each symbol only contains half the bits.

## 3. Results

### Channel Frequency Response

**Figure 8:**
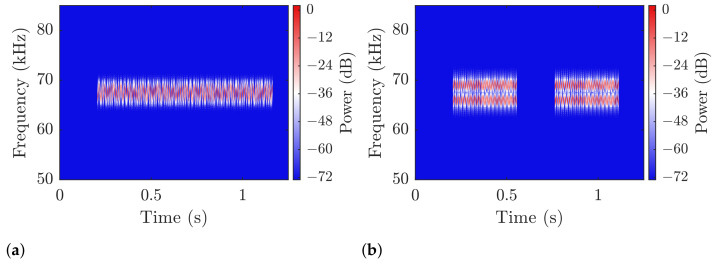

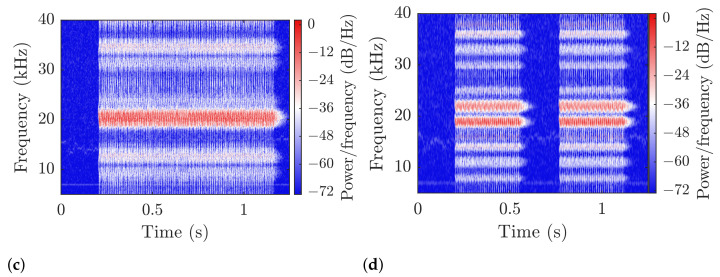
Spectrograms showing the intermediate frequency over time of parts of the signal to illustrate the effects of the channel and undersampling. (**a**) Single band signal after up-conversion before transmission; (**b**) Multi-band signal after up-conversion before transmission; (**c**) Single band after reception before down-conversion; (**d**) Multi-band after reception before down-conversion. Each package is transmitted three times in the experiment.

The transmitted and received signals are shown in Figure 8 as spectrograms over frequency and time. The undersampling introduces harmonic interference outside of the transmission band of the recorded signal, which are not physically present in the medium itself. Those phantom bands are removed on downsampling, by narrow bandpass filters.

**Figure 9:**
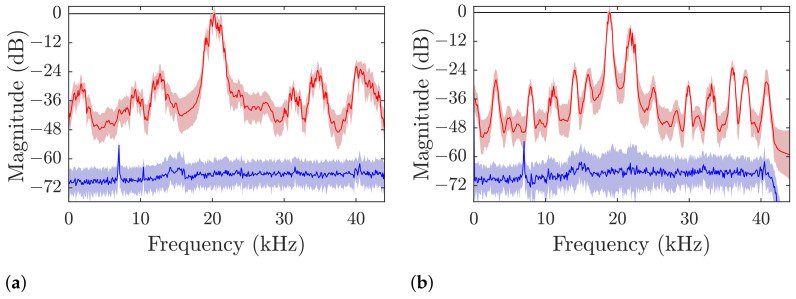
Averaged spectral power plots of the raw received signals. (**a**) Single band communication; (**b**) Dual-band communication. The colored area marks the ± 1 *σ* region of each frequency bin.

The power levels are more clearly visible in the averaged plots of Figure 9. The noise floor confirms the assumption of AWGN outside of the transmission, with an approximate SNR of 65 dB. The interference caused by the transmission itself raises the average power outside of the transmission band for about 30 dB.

## 4. Bit Error Rate and Packet Error Rate Simulations

The bit error rate (BER) and packet error rate (PER) of the proposed algorithms are estimated through simulation for an idealized channel as described in Section 2.3. We define the BER in two ways: By comparing each bit in the order of demodulation through the exclusive or (XOR) operation

(45) $$r_{\mathrm{BE}}=\sum_{n}\left(d_{\mathrm{est}}⊕d\right)+\left|N_{\mathrm{est}}-N\right|,$$

and by cross-correlation (XCorr), which returns the maximum match between the transmitted sequence *d* and demodulated sequence $d_{\mathrm{est}}$

(46) $$r_{\mathrm{BExc}}=\max_{n}\left\{d_{\mathrm{est}}⊛d\right\}+\left|N_{\mathrm{est}}-N\right|,$$

both of which include differences in the number of bits to account for additional or missing bits. The former (XOR) we regard for data transmission, where the content of the sequence is not known at the receiver, while the latter (XCorr) indicates the performance, if a known set of codes is expected. The PER is defined through the relative number of erroneous packets compared to the total number of sent packets, where a packet error is any packet that includes at least one bit error. For the PER we consider bit errors according to (45).

The probability of errors approximately follows the error function (*erfc*) over the SNR [63]. While there are closed-form approximations for LoRa [62,64], to ease comparisons we approximate those through superposed error functions

(47) $$P_{\mathrm{be}|\mathrm{pe}}\left(\gamma \right)=\sum_{q}\left(A_{q}\;\mathrm{erfc}\left\{{\left(B_{q}\;10^{\gamma /10}\right)}^{1/2^{q}}\right\}\right),$$

which were fitted manually for the coefficients in Table 3 and Table 4 for the BER and PER simulation as shown in Figure 11, respectively.

## 5. Discussion

**Figure 10:**
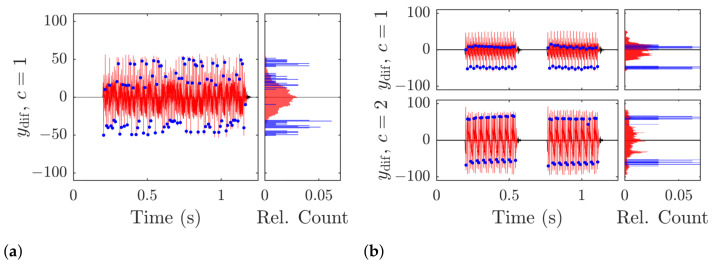
Time domain plot of the detected frames (red) and estimated symbols (blue dots). (**a**) Single band communication; (**b**) Dual-band communication. The symbol difference is not optimally detected, as the amplitude of the signal exceeds the amplitude of the estimated symbols. The histograms to the right of each time plot are normalized by the total number of samples (red) and symbols (blue) in each frame.

The transmissions in both scenarios (see Figure 10) were demodulated without error in the single and dual-band verification runs. The verification only cover a small range and is not meant to be exhaustive for a characterization of the system. The first point to notice is the relative lack of noise in the signal, which is illustrated by the blue background in Figure 8 over 60 dB below the highest signal levels. If we closely inspect the right edge of the plots in the lower row Figure 8c,d, the long ring-down of the signal spanning over more than 100 ms is visible as a brighter colored leg smearing the stronger power bands in time. This reminiscence of the signal in the channel affects the transmission as inter-signal interference and is the strongest cause of error in our transmission. The channel impulse response itself depends on the geometry of the body of water and the environment conditions, which are not covered by this investigation. However, the general behavior of the proposed communication scheme will hold in similar environments, and improve for less challenging conditions. The phantom bands that appear for higher and lower frequencies around the transmission are caused by the undersampling on reception and are not present in the physical channel. They are removed in the DDC by a narrow bandpass-filter and only will cause the signal to be corrupted, if one of the phantom bands overlap with the intermediate band where most of the signal power is shifted to. When we compare the frequency spectrum in and outside of symbols in Figure 9, the high signal-to-noise ratio becomes more obvious: The noise floor is at approximately 65 dB below the highest signal levels (blue lines), while the interference raises the floor to about −40 dB (red line, outside the intermediate bands).

If we regard the distributions of the symbols in Figure 10 around the decision threshold, which is at $y_{\mathrm{dif}}=0$, the single band levels show a multi-modal behavior. In the dual-band case, the distributions in all channels are much closer to uni-modal distributions, which is the ideal case, but are still far from the optimal. Optimal symbol levels would be achieved, if there would be two symmetrical probability bands at the minimal and maximal edge of the differential signal levels. This effect is due to the symbol peaks of up and down chirps not aligning in phase, but are shifted to each other. The symbol synchronization in the superposed signal locks-in on the matched filter output $y_{\mathrm{fhx}}$ that shows the strongest mean correlation, therefore pushing the other symbols out of phase. An additional phase offset correction before the summation of both matched filter outputs, i.e., before the JDS block, would move the secondary symbol levels closer to their ideal state and increase the overall performance, but is not included at this point. The general feasibility of our approach is thereby shown, but further practical verification is required.

**Figure 11:**
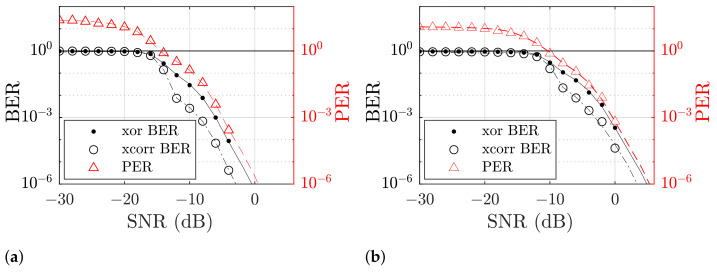
Plots of the simulated bit error rate (BER) and packet error rate (PER) for both single band (**a**) and dual-band transmission (**b**). The markers indicate each simulated SNR condition, the lines are manually fitted curves.

If we regard the results in Figure 11, the simulation of the transmission error probabilities through BER and PER does not include the multi-path response and, therefore, describes the idealized performance of the algorithm at that point. The approximate far field fading of acoustic signals in open water of approximately 20 dB km^−1^ in seawater can be used to design the acoustic output to suitable levels, e.g., for a BER of up to 0.1% over a range of 2000 m, the overall channel losses of approximately 40 dB have to be countered by about a combined gain of transmitter and receiver of 33 dB in the single band case and 39 dB in the dual-band case. More generally, the dual-band transmission is shifted by approximately 6 dB, which confirms the assumptions from (44) that a reduction of the bandwidth *B* to half the single band value also halves the symbol power $E_{\mathrm{sym}}$. The addition of a channel impulse response with additional echoes is expected to increase the error probability for all scenarios, as it introduces additional interference.

The PER follows the behavior of the BER, which is due to the definition that a packet is considered erroneous as soon as a single bit is demodulated wrongly. The error curves’ behavior for lower SNR to approach values over $10^{1}$ may be counter-intuitive for synchronized transmissions or when the number of symbols in a packet is known. Since we allow for arbitrary lengths and have no additional synchronization, for those conditions more packets are detected than were initially sent. This implies very short or fragmented packets, which can be disregarded on a higher level, if an additional protocol is implemented, e.g., that restricts the syntax of valid packages as in the NMEA 2000 standard of the National Marine Electronics Association [65].

If we regard the initially set application of acoustic communication in reverberating environments, the validation runs have shown that we can successfully transmit data even inside a very small body of water, while the simulation hints at the performance for larger ones, especially lakes and harbor areas, where there is little surf and most interference stems from ship engines. The change of acoustic properties in salt-water compared to fresh water is proportional to the length of the signal propagation, therefore for close proximity communications negligible, but requires consideration for long distance links. Hence, we assume the results of the pool runs can be extended to application at sea, lakes or oceans, albeit not as an accurate performance indicator for open water communication, but rather a worst-case scenario, in a highly reverberating environment, e.g., if two maintenance divers try to communicate in bad sight conditions near the ground or close to a ship’s hull.

## 6. Conclusions and Future Works

The proposed demodulator for preamble-free Chirp Slope Keying was implemented and the complete signal chain simulated and tested inside a measurement pool in a laboratory scale experiment for transmission rates of 100 bit/s for single band communications, as well as 200 bit/s for dual-band communication. The available bandwidth and symbol length was kept constant. The achieved BER estimated through the bitwise xor operation was simulated to drop below 0.001, i.e., 0.1% for SNR above −6 dB for a TB of 50 in the single band mode and for SNR above 0 dB for a TB of 25 in dual-band mode. The correct detection of packages and the demodulation was successfully implemented, verified and simulated as well. The PER follows the BER with an SNR offset of approximately 1 dB. The simulated channel contained Rayleigh fading and set the SNR through Additive White Gaussian Noise. A model for fitting the simulation results and parameters were disclosed, and required extensions for a more realistic simulation model discussed. The approach removes the necessity of preambles for multi-band communication that consume the limited available time slots. While the achieved data rates are low compared to narrow band communication schemes, the feasibility in a highly reverberating water tank has been shown, where those schemes will tend to exhibit high error rates if the preamble is not found. Equalization techniques can be employed to achieve better bit and packet error rate for lower signal-to-noise ratio, together with the design of specific RAKE devices to mitigate the multi-path characteristic of the underwater environment. We also strongly believe that investigations on the animals’ underwater hearing behaviour have to be done in order to better estimate the impact of artificial noise on the underwater environment. Further investigations can include the issues arising from the multiple access to the medium and in situations, where oxygen bubbles coming from the divers equipment are disturbing the channel.

## Figures and Tables

**Table 1:** Transmission Band Parameters.

Parameter	Value	Description
$f_{c}$	67.5 kHz	Center frequency
$\hat{B}$	5.0 kHz	Maximal available bandwidth
$f_{s}$	88.0 kHz	Receiver sampling frequency
$N_{\mathrm{res}}$	4	Resampling factor after down-mixing
$N_{\mathrm{res}2}$	2	Resampling factor after signal merge
$f_{s1}$	22 kHz	Sampling frequency after 1st downsampling
$f_{s2}$	11 kHz	Sampling frequency after 2nd downsampling

**Table 2:** Experiment Waveform Parameters.

Parameter	Value		Description
	*Single*	*Dual*	
*N*	96	64	Transmitted bits
$N_{\mathrm{lo}}$	1	2	Number of sub-channels
$N_{\mathrm{frm}}$	3	3	Number of packages sent
*B*	5.0 kHz	2.50 kHz	Bandwidth per channel
*T*	10 ms	10 ms	Length of a single chirp in time
$f_{c}$	67.5 kHz	[66.25, 68.75] kHz	Frequency offset to band center
TB	50	25	Time-bandwidth product

**Table 3:** Single band BER fit coefficients.

*q*		−1	0	1	2
$r_{\mathrm{BE}}$	$A_{q}$	0.95	−0.85	0.8	0.5
	$B_{q}$	26	50	22	140
$r_{\mathrm{BExc}}$	$A_{q}$	1.3	−0.5	0.8	0.02
	$B_{q}$	27	50	22	140
$r_{\mathrm{PE}}$	$A_{q}$	0	24	2.1	2.0
	$B_{q}$	0	50	25	140

**Table 4:** Dual-band fit coefficients.

*q*		−1	0	1	2
$r_{\mathrm{BE}}$	$A_{q}$	0.60	−0.65	0.65	0.65
	$B_{q}$	9.5	50	6.8	43
$r_{\mathrm{BExc}}$	$A_{q}$	1.00	−0.65	0.15	1.00
	$B_{q}$	10	22	10	65
$r_{\mathrm{PE}}$	$A_{q}$	4.0	6.0	1.8	1.2
	$B_{q}$	25	13	7.1	43

## Data Availability

The data presented in this study are available on request from the corresponding author.

## Abbreviations

The following abbreviations are used in this manuscript:

AF	Active Filter
AWGN	Additive White Gaussian Noise
BB	Baseband
BER	Bit Error Rate
BPF	Bandpass Filter
CSS	Chirp-Spread Spectrum
CSK	Chirp Slope Keying
DDC	Digital Down-Converter
DUC	Digital Up-Converter
FDDS	Frame Detect & Downsample
FrFT	Fractional Fourier Transform
FHX	Fast Hilbert Cross-Correlator
FSK	Frequency Shift Keying
JDS	Join & Downsample
LFM	Linear Frequency Modulation
LPF	Lowpass Filter
MUX	Multiplexer
PA	Power Amplifier
PER	Packet Error Rate
RX	Received or Receiver
SNR	Signal-to-Noise Ratio
TB	Time-Bandwidth Product
TX	Transmitted or Transmitter
UAV	Underwater Autonomous Vehicle
XOR	Exclusive Or Operation
XCorr	Cross-Correlation

## Appendix A

**Algorithm A1:** Linear Chirp Generation.
**input :** $f_{0}$ start frequency, *T* length of chirp in seconds, $f_{1}$ end frequency, $f_{s}$ sampling frequency. **output**: *y* 1-D vector containing a real-valued linear chirp sequence // Translate frequency input into relative frequencies: **1** $\omega_{0}=2\;\cdot \;\pi \;\cdot \;f_{0}/f_{s}$; **2** $\omega_{1}=2\;\cdot \;\pi \;\cdot \;f_{1}/f_{s}$; // Prepare sample time: **3** $N=\left\lfloor T\;\cdot \;f_{s}\right\rfloor$; **4** $n=0:\left(N-1\right)$; // Calculate the shaping window **5** $y_{w}=\mathrm{tukey}\left(N,\;0.5\right)$; // The discrete sampled time limited chirp signal is calculated as follows: **6** $k=\left(\omega_{1}-\omega_{0}\right)/2\;\cdot \;N_{\mathrm{ch}}$; **7** $\omega =\omega_{0}+k\;\cdot \;n$; // Furthermore, lastly the shaping function is simply superposed: **8** $y=y_{w}\;\cdot \;sin\left(\omega \;\cdot \;n\right)$;

**Algorithm A2:** Partially Constructed Tukey Window.
**input :** *N* length of window in samples, *a* taper fraction. **output**: *y* 1-D vector containing a real-valued window sequence // Prepare taper thresholds: **1** $N_{\mathrm{th}}=\left\lfloor a\;\cdot \;\left(N/2-1\right)\right\rfloor +1$; // Calculate 1st leg: Up slope **2** $n_{I}=0:\left(N_{\mathrm{th}}-1\right)$; **3** $y_{I}=\left(1-\cos\left(n_{I}\;\cdot \;\pi /\left(N_{\mathrm{th}}-1\right)\right)\right)/2$; // Calculate 2nd leg: Flat top **4** $n_{\mathrm{II}}=N_{\mathrm{th}}:\left(N-N_{\mathrm{th}}-1\right)$; **5** $y_{\mathrm{II}}=\mathrm{ones}\left(1,\;\mathrm{length}\left(n_{\mathrm{II}}\right)\right)$; // Calculate 3rd leg: Down slope **6** $N_{\mathrm{off}}=\left(N-1\right)\;\cdot \;\left(1-a/2\right)$; **7** $n_{\mathrm{III}}=\left(N-N_{\mathrm{th}}\right):N$; **8** $y_{\mathrm{III}}=\left(1-\cos\left(\left(n_{\mathrm{III}}-N_{\mathrm{off}}\right)\;\cdot \;\pi /\left(N_{\mathrm{th}}-1\right)\right)\right)/2$; // Join legs: **9** $y=\left[y_{I},\;y_{\mathrm{II}},\;y_{\mathrm{III}}\right]$;

**Algorithm A3:** Digital Up-Conversion.

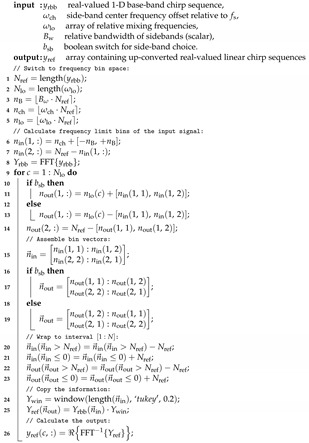

**Algorithm A4:** Digital Down-Conversion.

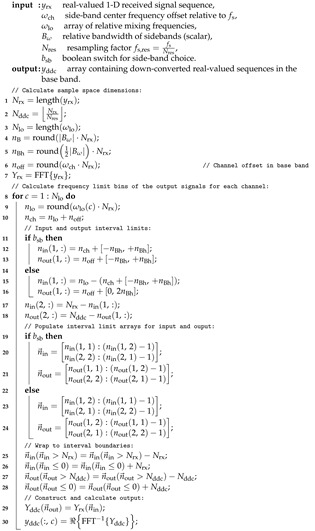

**Algorithm A5:** Chirp Slope Keying Modulator.
